# Supplementary material for: Coumarin Derivatives as New Toxic Compounds to Selected K12, R1–R4 E. coli Strains
Source: Materials (Basel). 2020 May 30;13(11):2499. doi: 10.3390/ma13112499 (PMC7321437; doi:10.3390/ma13112499)
Supplement: Supplementary file 1 [file materials-13-02499-s001.pdf]

materials

*Supplementary materials*

# Coumarin Derivatives as New Toxic Compounds to Selected K12, R1–R4 *E. coli* Strains

**Paweł Kowalczyk [1,*], Arleta Madej [2], Daniel Paprocki [2], Mateusz Szymczak [3] and Ryszard Ostaszewski [2]**

[1] Department of Animal Nutrition, The Kielanowski Institute of Animal Physiology and Nutrition, Polish Academy of Sciences, Instytucka 3, 05-110 Jabłonna, Poland
[2] Institute of Organic Chemistry, Polish Academy of Sciences, Kasprzaka 44/52, 01-224 Warsaw, Poland; arleta.madej@icho.edu.pl (A.M.); dpaprockizolw@gmail.com (D.P.); ryszard.ostaszewski@icho.edu.pl (R.O.)
[3] Department of Molecular Virology, Institute of Microbiology, Faculty of Biology, University of Warsaw, Miecznikowa 1, 02-096 Warsaw, Poland; mszymczak@biol.uw.edu.pl: ORCID: 0000-0003-4056-7792
* Correspondence: p.kowalczyk@ifzz.pl: ORCID: 0000-0003-4025-3750

Received: 23 April 2020; Accepted: 27 May 2020; Published: 30 May 2020

| Number in the Table | Compound | Molar Mass [g/mol] |
|---|---|---|
| 1 | 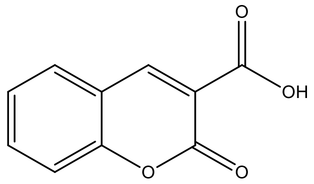 | 190.15 |
| 2 | 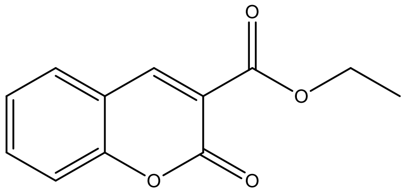 | 218.21 |
| 3 | 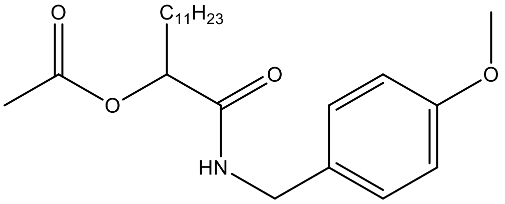 | 391.54 |
| 4 | 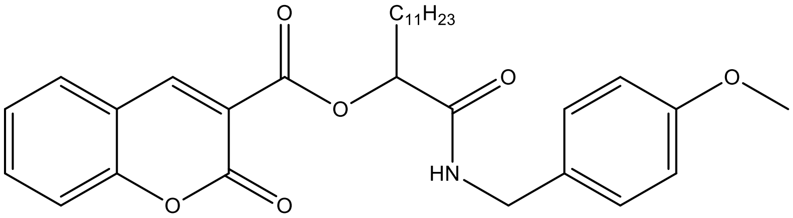 | 521.64 |
| 5 | 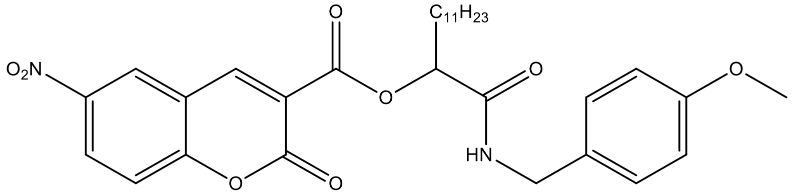 | 566.64 |

6
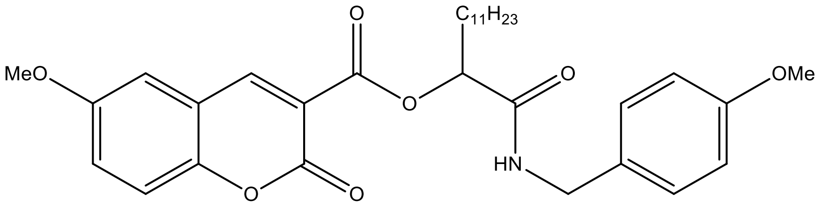

551.67

7
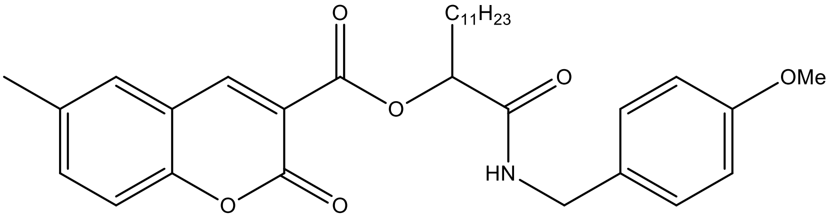

535.67

8
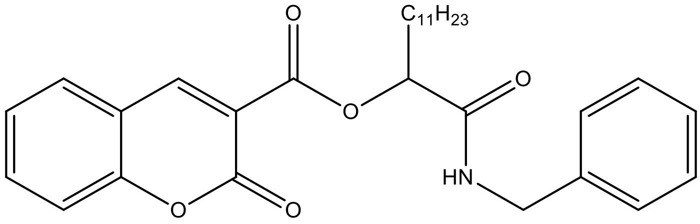

491.62

9
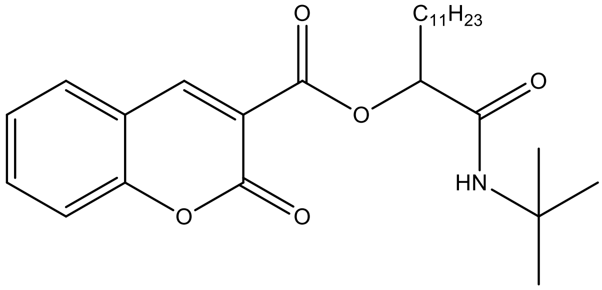

457.60

10
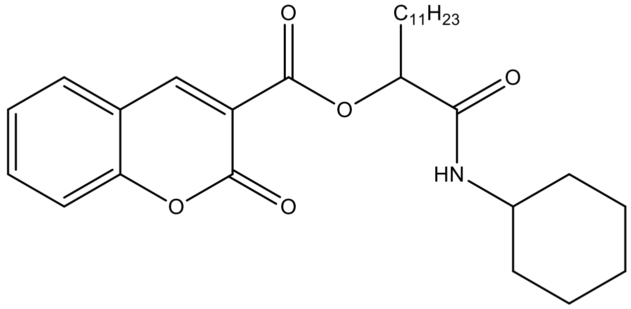

483.64

11
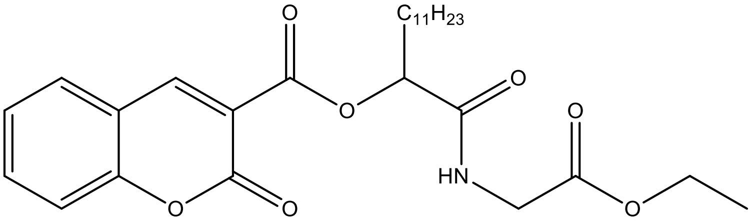

487.59

12
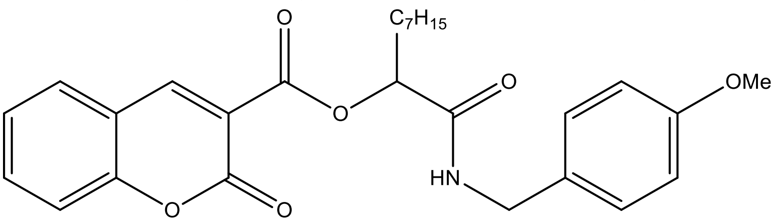

465.54

13 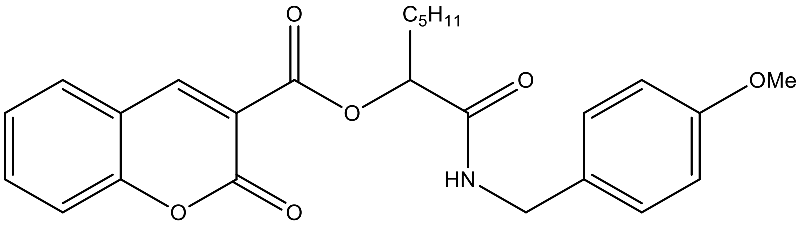 437.48

14 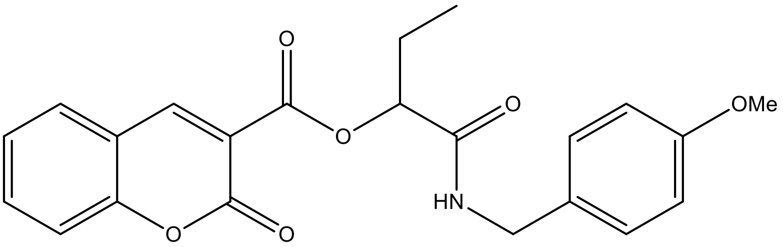 395.41

15 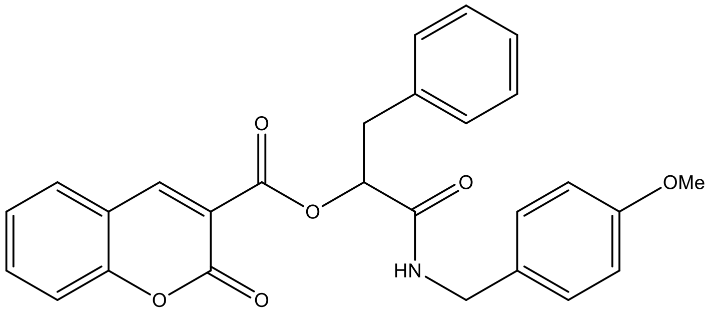 457.47

16 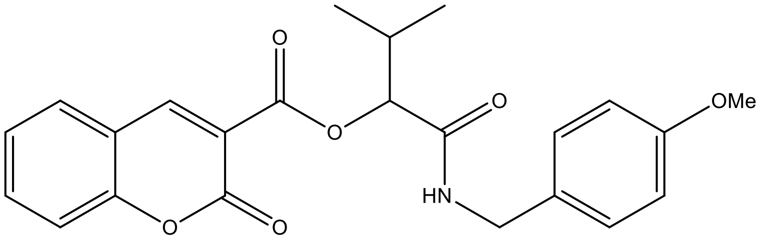 409.43

17 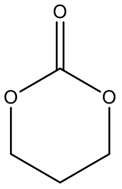 102.09

**Figure S1.** Structural formulas of coumarin derivatives.

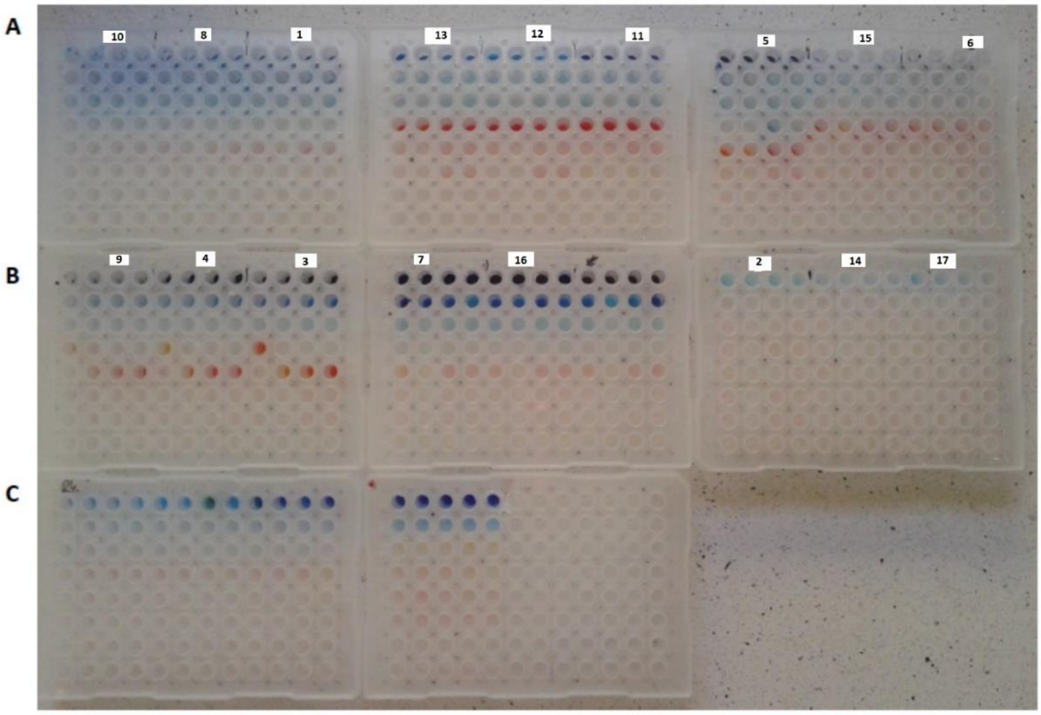


**Figure S2.** An example of the analysis of test compounds of various concentrations using a resusarin dye on microplates (mg $L^{-1}$). Panel A strains K12, R1, R2, R3 (with tested first 1–9 compounds). Panel B strains K12, R1, R2, R3 with tested 8 compounds (from 10 to 17). Number 18-control dilution. Panel C R4 strain with all 17 peptidomimetics. The order in which the compounds were applied to the plate was as follows in Table 1.
